# An un-commissioned randomized, placebo-controlled double-blind study to test the effect of deep sea fish oil as a pain reliever for dogs suffering from canine OA

**DOI:** 10.1186/1746-6148-8-157

**Published:** 2012-09-06

**Authors:** Anna Hielm-Björkman, Johanna Roine, Kari Elo, Anu Lappalainen, Jouni Junnila, Outi Laitinen-Vapaavuori

**Affiliations:** 1 Faculty of Veterinary Medicine, Department of Equine and Small Animal Medicine, University of Helsinki, P.O.Box 57, Helsinki, FI-00014, Finland; 2 Faculty of Agriculture and Forestry. Department of Animal sciences, University of Helsinki, P.O.Box 28, Helsinki, FI-00014, Finland; 3 4Pharma Ltd., Metsänneidonkuja 6, Espoo, FI-02130, Finland

**Keywords:** Osteoarthritis, Pain, Fish oil, Omega-3, Fatty acid, Canine / Dog, Supplement, Joint, RCT, Double-blind

## Abstract

**Background:**

An un-commissioned randomized, double-blinded, placebo controlled clinical study was planned using a deep sea fish oil product for pets. Seventy-seven client-owned dogs with osteoarthritis were randomly assigned to supplement the food with either the fish oil product or corn (=placebo) oil. Our main outcome variables were force platform variables peak vertical force (PVF) and impulse, the validated Helsinki Chronic Pain Index (HCPI) and the use of rescue NSAIDs. Secondary outcome variables were a locomotion visual analog scale (VAS), a Quality of life VAS, a comparative questionnaire, a veterinary assessment, owners’ final assessment of outcome and guessing the product given.

**Results:**

When comparing the two test groups at the end of the trial (16 weeks) there was no significant difference in any of the main outcome variables but owners of dogs that had taken fish oil were significantly happier with the treatment at the end visit and did significantly better at guessing what group their dogs had been in, compared to the placebo group. When comparing variables within the fish oil group as change from baseline to trial end, there were significant positive changes in PVF, HCPI, NSAID use, Quality of life VAS, as well as in all three scores in the comparative questionnaire (locomotion, every-day situations, and skin & coat). There were similar positive trends in force platform impulse and in the veterinary assessment variables, although they did not reach significance. Within the placebo group there were significant positive changes only in the HCPI and a significant deterioration according to veterinary assessment.

**Conclusions:**

When compared to placebo, there was not a major statistically significant benefit in using deep sea fish oil as a pain reliever in our study population of dogs suffering from osteoarthritis. However, the fish oil treated patients improved significantly in many of the variables, when comparing baseline values to the study-end values within the group, indicating a true but small relief in symptoms. Deep sea fish oil supplementation could be considered a part of the multimodal pain relieving approach currently recommended for dogs suffering from OA, especially for individuals that do not tolerate anti-inflammatory drugs.

## Background

Osteoarthritis (OA) is a major cause of chronic pain in dogs; it is estimated that 20% of the dog population in the United Kingdom and the USA are suffering from OA [1]. Canine hip dysplasia (CHD), elbow dysplasia (ED) and post cruciate ligament disease OA are three common forms of canine OA [2]. As OA rarely can be treated, it is kept silent primarily with non-steroidal anti-inflammatory drugs (NSAIDs), often being administered for long periods, even years. As these may have severe adverse effects, a recommendation has been made to use more natural disease-modifying agents in the pain management of OA, in animals and humans, alike [3,4]. To this end, more research is being conducted to find less detrimental treatments [5].

In human medicine it has been clear since the 80’s that fish oil helps people with rheumatoid arthritis (RA) [6-10]. The idea of treating canine OA with fish oils came from anecdotal evidence in the late 80s, where dogs in a trial of atopy, but with concurrent hip dysplasia, had been given fish oil for their dermatological problem and at the same time the owners reported a positive effect on their pets’ pain and lameness symptoms [11,12]. In spite of this, the first randomized, controlled, blinded canine fish oil trials on OA patients were published only in 2010 [13-16].

As the pathophysiological events associated with OA are becoming increasingly understood, anti-inflammatory eicosanoids are considered attractive new therapeutic disease modifying agents that target the inflammatory process [17]. The eicosanoids derive from three different essential fatty acid (EFA) cascades. The first, the omega(ω)-6 arachidonic acid (AA) cascade results in many pro-inflammatory eicosanoids such as leukotriene B_4_ and prostaglandin E_2_, where at least the latter has been shown to positively correlate with OA pain [18]. One of the other two parallel cascades that compete with the AA cascade is the ω-3 eicosapentaenoic acid (EPA) cascade, resulting in more anti-inflammatory eicosanoids [19-21]. The proposed anti-inflammatory working mechanisms of fish oil lies in their high content of acids from the last section of this EPA cascade, namely EPA and docosahexaenoic acid (DHA). Also in dogs it has been shown that by increasing the content of EPA or other oils from this particular cascade, there will be partial replacement of AA in cell membranes by EPA and DHA, which leads to a shift from the pro-inflammatory eicosanoids that the AA cascade forms to the anti-inflammatory eicosanoids the EPA cascade forms [22,23]. EPA is also known to have anti-inflammatory effects of its own; it may suppress the production of pro-inflammatory cytokines such as interleukin-1β and −6 and tumor necrosis factor-α [24-26]. Also, a significant reduction in activities of matrix metalloproteinase (MMP)-2 and −9, which contribute to cartilage destruction, as well as a significant increase in the tissue inhibitor of MMP-2 (TIMP-2) has been recorded after fish oil supplementation [27,28]. As dogs and cats in the western world nowadays primarily eat commercial foods, Remillard has even suggested that our animal patients with chronic inflammatory diseases might either be primary ω-3 deficient and/or ω-6 toxic, as commercial diets often have a very high ω-6:ω-3 fatty acid ratio [29].

The purpose of the study was to test if supplementing a basic commercial canine diet with liquid fish oil rich in EPA and DHA, would benefit patients already suffering from a chronic inflammatory process such as OA. We hypothesized that the dogs taking fish oil would show an increase in ground reaction forces, a decrease in the owner and investigator evaluated lameness and pain scales, a decrease in NSAID use, and a decrease in serum AA as well as an increase in serum ω-3 fatty acids, compared with a control group that got corn oil as a supplement.

## Results

### Dogs

Two hundred and thirty dog owners returned the application questionnaires. After an application screening, ninety-three owners were interviewed by telephone to get additional information and eighty-nine of these were then invited to the first screening visit.

**Figure 1:**
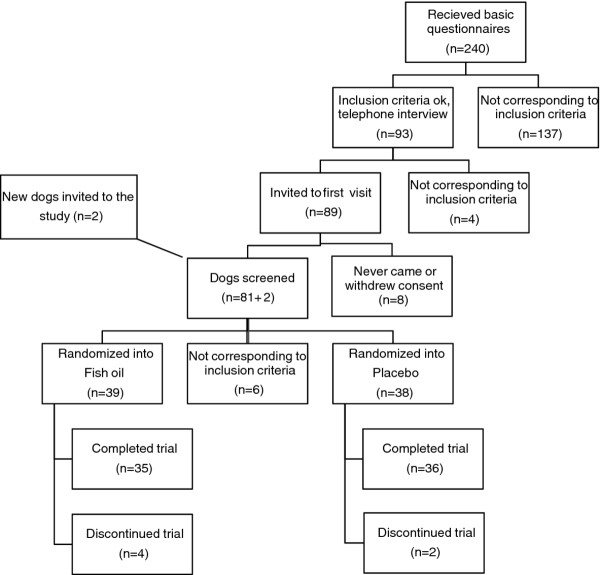
Flow diagram through study.

Six owners either never came to the baseline evaluation or withdrew their consent so two new dogs were invited for screening and passed. Eight dogs were excluded because of the following reasons: abnormal laboratory results (n = 4), a Helsinki Chronic Pain Index (HCPI) under 6 (n = 1), same grade lameness in two legs (n = 2) and not having OA (n = 1). Seventy-seven dogs continued to the medication phase. Six owners were lost to the follow-up; four from the treatment group [owners wanted to exit study because their dog was losing weight even after food change (n = 1), owner did not want to comply to the study plan (n = 1), owner did not get enough help for pain from the rescue NSAID (n = 1), owner opted for euthanasia (n = 1)] and two from the placebo [hemorraghic enteritis (n = 1), owner opted for euthanasia (n = 1)]. Seventy-one dogs finished the study; 35 dogs from the treatment group and 36 dogs from the placebo group (Figure 1). Of the 71 dogs there were 14 mixed-breed dogs and 30 different breeds represented: 6 Collies, 5 German Shepherd Dogs, Golden Retrievers, and Labradors, 4 Baucerons, Bernen Sennen Dogs, and Rottweilers and all other breeds with 1–3 dogs/breed. There were both uni- and bi-laterally affected dogs. All dogs had either moderate or severe radiological changes in the worst affected joint. At baseline (W_0_), eight dogs were still taking NSAIDs; five in the treatment group and two in the placebo group were taking 1–2 doses per month and one dog in the treatment group was taking 3–5 tablets per week. There was no baseline bias between the groups at the start of the trial (Table 1).

**Table 1:** Baseline characteristics of both treatment groups

**Baseline data**	**Fish oil**	**Placebo (corn oil)**	**P-value**
Total no of subjects (finished the study)	39 (35)	38 (36)	P = 1.000
Male / female	17/22	17/21	P = 1.000
Castrated / sterilized / intact	7/15/17	10/10/18	P = 0.427
Body condition score (median, total range 1–5)	3 (2–5)	3 (2–4)	P = 0.852
Mean bodyweight ± SD, kg	33.5 ± 12.0	34.2 ± 8.6	P = 0.755
Mean age ± SD, years	5.6 ± 2.9	6.5 ± 3.0	P = 0.227
Food eaten at study end (beef / chicken / other)	33/5/1	31/7	P = 0.961
Hip OA (only hip OA)	34 (17)	36 (14)	P = 0.431
Stifle OA (only stifle OA)	5 (1)	5 (0)	P = 1.000
Elbow OA (only elbow OA)	13 (1)	13 (1)	P = 1.000
Any OA + spondylosis or vertebra-OA	8	13	P = 0.208
Duration of symptoms (median)	over 2 years	1-2 years	P = 0.312
Mean HCPI score at inclusion (W_−2_) ± SD	16.6 ± 5.8	17.4 ± 5.8	P = 0.464
Number of High (>17) / Low (0–17) HCPI in group	18/21	18/20	P = 1.000
Peak vertical force at baseline ± SD	76.7 ± 19.6	77.2 ± 20.5	P = 0.918
Impulse at baseline ± SD	9.9 ± 3.5	10.0 ± 2.9	P = 0.909
Mean Lameness VAS at baseline ± SD	3.8 ±1.9	4.5 ± 1.9	P = 0.138
Mean Quality of life VAS at baseline ± SD	3.6 ± 1.8	3.8 ± 1.8	P = 0.712
Mean Veterinary index at baseline ± SD	2.4 ± 2.4	2.5 ± 2.2	P = 0.881
Median NSAID use, first questionnaire (W_−8_)	0-2 x/month	0-2 x/month	P = 0.317

P values are for comparison between groups: In 2 x 2 tables the exact Fisher’s tests were used, in nominal to ordinal/scale Linear-by-linear association Chi-Square test were used.

### Study foods and supplements

Nine dogs started with the sensitive rice/chicken food while 66 started with the basic wheat/beef food. Four more dogs changed their diet to the rice/chicken during the trial while two dogs changed food from rice/chicken to the basic. One dog started out on the basic, changed to rice/chicken and then changed to Frank’s Pro Gold, which it then ate through the rest of the study. The wheat/beef diet gave on average 0.018 mg ± 0.006 (SD) (range 0.01-0.04 mg) of ω-3 fatty acids per dogs’ kg BW and the rice/chicken diet was very similar. The fish oil gave 110.25 mg ± 5.75 (SD) predominantly EPA and DHA, and the corn oil 1.7 mg ± 0.09 (SD) (range 1.4-1.8), predominantly α-linolenic acid (ALA), of ω-3 fatty acids, per dogs’ kg BW.

Both foods and oils were palatable: 98.4% of the dogs that ate the basic food and 87.5% of the dogs that ate the rice/chicken food liked it; 91.4% of the dogs that ate the fish oil and 97.2% of the dogs that ate corn oil, liked what they were eating. There was no statistical difference in how dogs liked the foods and the oils.

When the owners evaluated appetite, vomiting, diarrhea and skin problems/itching every month, there were never any significant differences between the two groups. One dog from the placebo group suffered from hemorrhagic enteritis. The used oil had been stored in a refrigerator as instructed and was analyzed for contaminating bacteria but found negative. This dog was already on the sensitive dogs’ food as it had a history of easy diarrhea and the owner decided to leave the study.

Covariates are mentioned in the results only when they were significant.

### Main outcome variables

Differences between treatment groups were not significant for any of the four main outcome variables (force platform variables PVF and impulse, the HCPI and rescue NSAIDs). Results at baseline and end of trial per group, as well as change from baseline to end of trial both within and between groups for all variables are shown in Table 2.

**Table 2:** Primary and secondary variables per group at start and end of trial and P-values for change within and between groups

**Variable**		** Fish oil**			** Placebo**		**Between**
	**Baseline**	**End of trial**	P-value^*^	**Baseline**	**End of trial**	P-value^*^	**P-value**^†^
**PVF**	76.7 ± 19.6	82.6 ± 22.6	**0.021**	77.2 ± 20.5	79.3 ± 22.1	0.210	0.388
**Impulse**	9.9 ± 3.5	10.2 ± 3.5	0.092	10.0 ± 2.9	10.6 ± 3.4	0.207	0.699
**HCPI**	16.1 ± 5.4	12.8 ± 6.8	**<0.001**	17.1 ± 6.4	15.0 ± 6.9	**0.013**	0.335
**NSAID use**^‡^	1.0 ± 1.4	0.4 ± 0.8	**0.017**	0.7 ± 1.2	0.4 ± 0.9	0.214	0.740
**Locomotion-VAS**	3.8 ± 1.9	3.5 ± 2.1	0.489	4.5 ± 1.9	4.0 ± 2.2	0.220	0.354
**QOL-VAS**	3.6 ± 1.8	2.9 ± 1.8	**0.045**	3.8 ± 1.8	3.5 ± 1.9	0.301	0.749
**Comp. mobility**	26.1 ± 4.55	23.1 ± 11.0	**0.021**	25.9 ± 4.1	24.1 ± 5.7	0.122	0.525
**Comp. daily chores**	22.7 ± 4.7	19.2 ± 9.5	**0.011**	21.6 ± 4.2	20.2 ± 5.6	0.339	0.595
**Comp. skin/hair**	7.9 ± 1.1	6.9 ± 3.0	**0.025**	7.8 ± 1.6	7.5 ± 2.0	0.424	0.572
**Vet. index**	2.4 ± 2.4	2.1 ± 2.5	0.096	2.5 ± 2.2	2.7 ± 2.6	**0.009**	0.196
**Owner final (1–5)**^§^	NA	1.3 ± 1.2	NA	NA	1.9 ± 1.1	NA	**0.029**
**Group? (1–2)**^£^	NA	1.4 ± 0.5	NA	NA	1.7 ± 0.5	NA	**0.028**

^*^ = P-value of comparing start of trial to end of trial, within group.

^†^ = P-value of comparing change from baseline to end of trial, between groups, except for the last two (“owner final” and “Group?”), where the P-value is the difference between the two group means at end of trial.

^‡^ = W_−8_ was here used as baseline, as owners were asked to not use NSAIDs at W_0_.

^§^ = At the end of trial the owners answered a questions on how well the treatment corresponded to their idea of a successful treatment; 1 = corresponded fully, 2 = quite well, 3 = to some parts, 4 = not so well, 5 = not at all.

^£^ = Owners guessed that their dogs were in 1 = the treatment or in 2 = the placebo group.

Comp. = comparative questionnaire. Values P <0·05 are bolded. NA = data not available.

With the force platform variables the variation between dogs was notable. When looking at the mean values of the percentage change from baseline to end of study, no difference between treatment groups was present in either PVF or impulse. However, when looking at the change from baseline to end of study within the fish oil group there was a significant change (P = 0.021) for the PVF and a trend towards change in the impulse (P = 0.092). These changes were not significant in the placebo group.

Within both fish oil and placebo groups, the HCPI significantly decreased between baseline and end of trial (P < 0.001 and P = 0.013, respectively). For a reason unknown to us, the HCPI increased from baseline to week four in the fish oil group.

**Figure 2:**
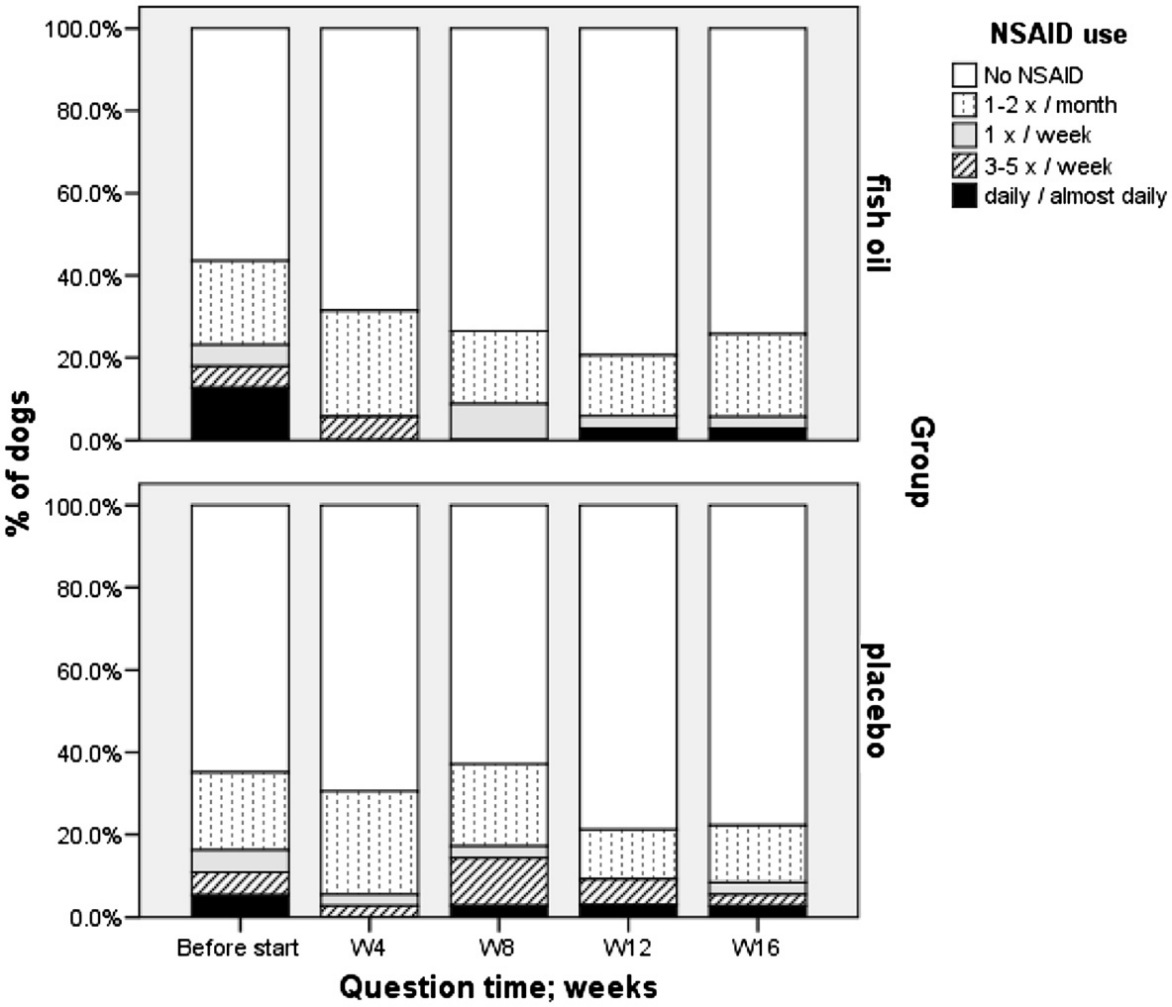
Use of NSAIDs before the start of the trial and use of NSAID rescue medication during the trial.

The use of NSAIDs decreased in both groups between W_−8_ and end of trial: e.g. NSAID given 3–7 days per week decreased in the fish oil group from 17.9 to 2.9%, and in the placebo group from 10.8 to 5.6%, respectively (Figure 2). However, only within the fish oil group, the decrease of rescue NSAIDs was significant (P = 0.017). NSAID use at 8 weeks before the study seemed to be significant in explaining NSAID use during the study: patients using more NSAIDs before the study were more likely also to use them during the study.

### Secondary outcome-variables

When comparing the opinions of the owners in the two groups at the end of trial, there was significant differences both in how owners considered the results to correspond to what they had hoped for as a good treatment and in which group they suspected their dog had been in (Table 2). Given as quantitative percentages; 65.7% of the owners of dogs in the treatment group were happy with the treatment compared to only 38.9% in the placebo group and 60.6% of the owners that were from the treatment group guessed that they were from the treatment group whereas only 31.4% of the owners that were from the placebo group guessed that their dogs were in the treatment group).

Within the treatment group, the change in the QOL-VAS from baseline to end of trial was statistically significant (P = 0.045), whereas this was not the case in the placebo group. Neither groups’ changes were significant for the lameness-VAS. Also here, the VAS-scores in the treatment group deteriorated between baseline and 4 weeks and only after that their condition improved; then at a higher speed than in the placebo-group.

All three comparative questions sum-scores were significantly better at the end of study compared to baseline in the treatment group: locomotion (P = 0.021), every-day situations (P = 0.011) and skin & hair coat (P = 0.025). These differences were not statistically significant in the placebo group. In the every-day-situation score no covariates significantly explained the change from baseline. In the comparative locomotion- and hair coat questions, back radiology was a significant factor in explaining differences in the sum of scores: Patients with more radiological findings in the spine also had higher scores in the movement and coat related questions.

Within the groups, the placebo dogs were assessed by veterinarians as having significantly deteriorated in lameness, jumping and walking stairs (P = 0.009). The treatment group had become better but this did not quite reach significance (P = 0.096). Although radiological findings in the stifle or thoracolumbar spine had some influence on the veterinary scores, the effects did not remain statistically significant when put together to form a final model.

### Serum analyzes

To test for owner compliance and to see that the product indeed had been ingested, the amount of EFAs in plasma phospholipids was analyzed. There was a significant increase in EPA from 1.0 ± 0.08 to 6.8 ± 0.5 (P < 0.001) and DHA from 1.5 ± 0.1 to 3.0 ± 0.2 (P < 0.001), and a decrease in AA from 19.5 ± 0.4 to 14.2 ± 0.4 (P < 0.001) in the fish oil group compared to a significant increase only in AA from 19.5 ± 0.4 to 20.2 ± 0.4 (P = 0.030) in the placebo group, indicating that the dogs had taken their proper products.

## Discussion

In evidence-based medicine, unbiased randomized controlled trials are crucial in the decision-making of which treatment to use. The results of this study suggest that there is some benefit in feeding an ω-3 fish oil supplement to dogs with OA, but that this benefit is relatively modest. There was no statistical difference between the fish oil and the placebo groups in any of the primary treatment effect variables, and only in two of six secondary outcome variables, being the owners’ final evaluation of the result and his guessing of the supplement given. Therefore our hypotheses of difference in treatment effect between treatment group and placebo group failed. However, there were several significant changes between baseline and end of trial especially in the fish oil group, indicating that there is some effect but that the effect is not strong. Previous research indicates that there could be an anti-inflammatory effect [17-21].

There are four recent canine fish oil studies; all four were randomized, controlled, blinded but all were also supported by an international pet food company [13-16]. Although there is no reason to suspect any industry bias we feel that it is important that also un-commissioned university based studies are conducted. We can, however, now conclude that our results were very similar to the previously reported canine OA studies. Roush et al. found 3/13 different variables (two at one single time out of 3 possible time points) and one at two time points, giving a significant change in 4/39 variables, possibly indicating a very small treatment effect – or statistical chance [13]. Their second study found no significant differences between treatment and placebo groups but one could see a slightly greater reduction of lameness over time, only within the treatment group, indicating a small effect [14]. The third study showed a small but significant difference in NSAID intake between groups, indicating a more curative effect of the ω-3 diet [15]. The fourth study found a significant (food over time) interaction effect in 2 of 5 of the outcome variables, between the food with least and most EPA + DHA (0.8% and 2.9%, respectively) [16]. So far there is no study evaluating only fish oil for OA in humans but there are three studies evaluating fish oil with something else for humans suffering from OA. One study using a product containing fish oils rich in ω-3 and ω-6 fatty acids, nettle (*urtica dioica*), zinc and vitamin E, showed a big significant decrease of both NSAID use and, pain, stiffness and function scores (WOMAC) in the treated group, compared to the placebo group [30]. The results were criticized in a letter to the editor where the writer pointed out that the results presented would have been 76% more effective for pain reduction than an intra-articular corticosteroid injection [31]. Another study evaluated fish oil combined with lemon verbena (*Aloysia triphylla, Lippia citriodora*) extract. Also here validated scores were used (WOMAC and Lequesne) and showed that the treatment reduced symptoms of pain and stiffness significantly and improved physical function both between groups and in the treatment group but not in the placebo group [32]. A third study compared glucosamine alone and glucosamine plus a combination of fish oil and other ω-3 oils, where the combined group gave significantly better results [33]. As the products used in the human studies were different from the ones used in the canine studies, it is of course possible that the added nettle, lemon verbena, zinc or glucosamine might have made these products superior. Anyway, results this strong have not yet been published for dogs.

Different factors such as heterogeneous patient material, ω-6: ω-3 ratio, EFA dose or duration of treatment may have decreased the effect size, if it existed. In our study population we found a variation in severity and location of the OA as well as co-morbidity like spondylosis of the vertebras and secondary lameness. It would be preferable that all patients only suffered from OA in a single joint in a single leg, especially when evaluated for ground reaction forces using a force platform. However, all dogs with OA start to shift weight, leading to secondary lameness in its other legs that at times can be even worse than the initial “worst” leg. Front leg secondary tendinitis or muscle cramps can also introduce noise into gait evaluations. This could be seen in the large standard deviations of the force platform variables in our results that could not be explained by treatment group or any of the considered covariates. As fish oil has been shown to work on inflammatory factors, it is possible that the supplement will not work on secondary non-inflammatory pain such as from osteophytes, trigger points or cramps. This will always be the downside of conducting clinical trials, in humans and dogs alike. On the other hand, the upside is that heterogeneous group of patients mimics the real clinical situations where we treat; the patients do not all suffer from the same induced arthritis but have concurrent back problems, more than one leg or joint is involved and they are not all the same size and age. In times of ethical awakening and with translational pain research emerging, it is also much more ethical and clinically relevant to use owner owned dogs that have the real, chronic disease and that live a normal life with their families, than laboratory dogs with induced arthritis.

Our serum analyses showed that the AA decreased and the EPA and DHA increased in our fish oil group, whereas no such change could be seen in the placebo group, confirming that dogs had ingested the right oils. Such changes have been reported repeatedly in dogs after ingesting fish oil formulas [13,23,27,28,34] even though the decrease in AA proportion has not been seen in all human studies [21].

In the present study the ω-6:ω-3 ratio for the fish oil was 1:14, for the corn oil it was 52:1 and in the dog food 11:1. The ratio of ω-6 to ω-3 was initially believed to be important [35] but has now been shown to be of less importance [23]. Nowadays it is recognized that it is the absolute dose of the ω-3 and the ω-3 source used that are more important, as the conversion rate differs depending on the oil - e.g. much more vegetable ω-3 ALA is needed for the ineffective conversion of ALA into EPA than if you give fish oil EPA directly [23,27]. It has both been suggested that it would be better to give more DHA than its shorter precursors, eg. EPA [34], as well as to give mainly EPA instead of DHA, especially for OA patients [36]. But, as both EPA and DHA have anti-inflammatory properties of their own and as they both come out when fish is pressed, it might be useful to give them in combination. Different ratios could be tested for effect in further studies.

It is conceivable that a larger dose of the fish oil could have given a better result. The dose of the ω-3 oils used in the present study (90 mg EPA, 20 mg DHA and 5.4 mg ETA per kg BW) was, however, similar to the doses that have been used in other studies: In human adults daily doses of 1.8-3.2 g of EPA and 0.9-2.4 g of DHA were used in studies for different inflammatory diseases [37-39]. If a human is considered to weight about 70 kg, the kg calculated daily doses from these studies would be between 26–45 mg EPA and 13–34 mg DHA per kg BW. In one canine study the researchers wanted to try a DHA enriched fish oil formula and used 11–14 mg of EPA and 17–21 mg of DHA per kg BW [34]. Another canine study used 48 mg of EPA and 42 mg of DHA per kg BW [28]. Other studies give the amount of ω–3 fatty acids as g per kg food. The Hill’s studies give the ω-3 dose as % of food on a dry-matter basis and conclude that the test food had 3.47% ω-3 s and the placebo food 0.11%, meaning that the test food had an over 31-fold higher amount of total ω-3 s, compared to the control [13]. Hall used 6.2 g ω-3 fatty acids per kg of food [23]. As we do not know how much the dogs ate in that study we can, however, not calculate the mg/kg BW daily doses. Blonk showed that, in humans, doses over ~1.2 g DHA/day (given as fish oil) saturates the plasma DHA concentration and further increase of given DHA increase the plasma concentration only incrementally [40]. This has not to our knowledge been studied in dogs. Hall concluded that 175 mg of DHA/kg BW/day was needed to attain maximum plasma levels of DHA in dogs [23]. As EPA will change into DHA in the EPA cascade, it is however unclear how much of EPA and DHA combined is needed to achieve maximum plasma levels. In fish oil the EPA concentration is usually about 60% and the DHA 40% [41]. In the product we used the DHA was about 22% of the EPA amount. Although we had a significant increase in both DHA and EPA in our end-of-trial samples in the fish oil group, it is possible that we did not come up to the maximum plasma levels. Therefore we cannot say if a bigger dose still would have benefitted the outcome. However, as our oil contained only fish derived EPA and DHA and as there was a big difference in the ω-6:ω-3 ratio between the fish oil and the placebo, we think that our dose and ratio used led to at least some positive results. As EPA, DHA and other EFAs from the EPA cascade have been shown to have anti-inflammatory properties, this supports the clinically positive, although mild, results seen in our treatment group and not in the placebo group.

Our study period was 16 weeks. Less than 12 weeks have been considered too short in human studies [42]. We could see a small worsening in the treatment group at week four based on HCPI and the VASs. This has not been reported before. It is hard to say why this worsening occurred, as the owners otherwise did not comment on anything being negative in the same time period. After this the pain and lameness seemed to decrease and QOL to increase at a slightly higher rate in the treatment group than in the placebo group, so without the sudden exacerbation at week four or with a longer follow-up, a significant difference between treatment group could have been achieved favoring the treatment group. However, with the data at hand, no significance difference could be detected.

## Conclusion

When compared to corn oil (placebo), there was not a statistically significant benefit in using deep sea fish oil as a pain reliever in our study population of dogs suffering from osteoarthritis. However, when comparing baseline values to the study-end values within the group during the 16 week study, the fish oil treated patients improved significantly in many of our outcome variables indicating a true, but small, relief in pain symptoms. During the same time there was nearly no effect within the placebo group. As other studies have shown a similar effect, we feel we can recommend fish oil e.g. for patients that cannot take NSAIDs or other analgesics for their OA.

## Methods

### Study protocol

The study was designed as a randomized double-blind clinical trial with a treatment group and a negative (=placebo) control group, using the CONSORT guidelines [43]. Owners were asked not to give their dogs NSAIDs or corticosteroids for 15 days prior to the study and not to use Na-pentosan polysulphate (Carthrophen®, Biopharm Pty. Ltd., Australia) for 30 days prior to the study.

The outline of the study can be seen in Table 3. All dog owners that contacted us first completed a questionnaire. Based on this and a following telephone interview conducted by a secretary blinded to the randomisation list, dogs were invited to the pre-baseline screening visit, two weeks before the baseline (W_−2_). At this screening visit the dogs were assigned into groups in order of arrival using a computer-generated 4 block random list. A high (over 17) or low (17 or lower) level of pain according to the Helsinki chronic pain index (HCPI) [44-46]^,^ and normal or sensitive diet were stratified for. The owners answered a second questionnaire and dogs were evaluated by a veterinarian, they were customized to the force platform and blood samples were taken and analyzed for inclusion or exclusion. All dogs were sedated using medetomidine chloride (Dorbene®, Vetcare Oy, Salo, Finland) at 20–80 μg/kg BW and propofol (Propoclear®, Scanvet, Parola, Finland) at 2 mg/kg BW and radiographs were taken of the dogs’ hips, lateral lumbar spine, knees and elbows, and other joints if needed. Dogs were excluded from the trial if either blood values or radiographs showed that they were not eligible for the study. All dogs started the new diet after this first visit.

**Table 3:** Timeline: Products given and questionnaires, tests and evaluations done during study period

**Time**	**Questionnaires and visits**	**Food, rescue analgesia and tested products**	**Outcome variables, other tests and evaluations**	
			**Questionnaires**	**At clinic**
**W**_**−8**_	Internet / posted questionnaire		Descriptive data. Questionnaire on pain items, including HCPI. Lameness and QOL VAS. Use of NSAIDs / adjuvant analgesics.	
**W**_**−8**_**to W**_**−2**_	Telephone interview		Questions about unclear answer in the inclusion questionnaire	
**W**_**−2**_	Screening visit	New feed started.	Questionnaire on pain items, including HCPI. Lameness and QOL VAS. Use of NSAIDs / adjuvant analgesics.	Thorough veterinary evaluation + lameness, jumping and stairs on video. PVF, Impulse. Blood samples (inclusion). Radiographs.
**W**_**0**_	Baseline visit	Oils started. Rescue NSAID given to owner.	Questionnaire on pain items, including HCPI. Lameness and QOL VAS. Use of NSAIDs / adjuvant analgesics.	Veterinary evaluation: Lameness, jumping and stairs on video. PVF, Impulse. Blood samples.
**W**_**4**_	4 weeks after baseline		Questionnaire on pain items, including HCPI. Lameness and QOL VAS. Use of rescue analgesic. Adverse effect questions. Comparative questions: locomotion, every-day situations and skin and hair-coat.	
**W**_**8**_	8 weeks after baseline		Same as at W_4_.	
**W**_**12**_	12 weeks after baseline		Same as at W_4_.	
**W**_**16**_	End visit (16 weeks after baseline)	Feed and oils stopped.	Questionnaire on pain items, incl. HCPI. Lameness and QOL VAS. Use of rescue analgesic. Adverse effect questions. Comparative questions: locomotion, every-day situations and skin and hair-coat. Final assessment of outcome. Guessing group.	Veterinary evaluation: Lameness, jumping and stairs on video. PVF, Impulse. Blood samples.

There were two evaluation visits to the clinic: the baseline visit (at week zero = W_0_), after which the dogs started with the product or the placebo and the “end of trial”-visit (at week 16 = W_16_), when products had been taken for 16 weeks. For ethical reasons all owners were also given an NSAID that could be used in case of severe pain; 10 tablets of 227 mg firocoxib (Previcox®, Merial Norden A/S, Denmark) in normal packaging, with dosing instructions (5–10 mg / kg BW, once daily). More of this rescue medication was supplied upon request during the whole study period. At both evaluation visits the owners answered questionnaires, the dogs were evaluated by a veterinarian and tested on gait analysis equipment, and blood samples were taken.

All evaluators (veterinarians and owners) and all technical assistants were blinded. Owners of the dogs were required to sign informed consent forms. They could drop out of the study without giving a reason at any time. The study protocol was approved by the Ethics Committee of the University of Helsinki and the study was conducted at the Small Animal Hospital of the University of Helsinki.

### Dogs

Dogs were recruited for the study through advertisements in daily papers, dog magazines, pet stores, veterinary clinics and dog parks. Patients were considered eligible for inclusion if they had clinical signs and a radiographic diagnosis of hip, elbow and/or knee osteoarthritis. The owner had to describe at least three of the following signs as being frequent: difficulty in lying down and/or in getting up from a lying position, difficulty in jumping or refusing to jump, difficulty in walking up or down stairs, or definite lameness. The dogs had to be over 18 kg (because of the force platform analysis), over 1 year of age and have a HCPI of more than 6. Exclusion criteria were if the dogs were pregnant or lactating, had neurological deficits, lameness from acute articular infection, recent trauma or, if the owner would not comply with changing the dog’s diet and/or give the supplement daily. All dogs lived with their owners during the whole study period and came in only for the evaluations. One change in eligibility criteria was made already before baseline: As some owners felt they could not leave out the NSAIDs two weeks prior to the study it was decided that these dogs anyway should be left in the study. Also, as owners were told not to give NSAIDs or other analgesics two weeks prior to the baseline visit, we could not use the “NSAID use at the baseline visit” as a prior to trial medication variable; instead we used the info on NSAID use that we received from the owners the first time we asked them, at W_−8_. The used variable would therefore be the change in NSAID use between the first questionnaire and the trial-end visit.

### Food and test products

There were two commercial foods available, either a basic wheat/beef (Royal Canin® Croc) or a rice/chicken sensitive formula (Jahti & Vahti® lamb and rice) dry dog food. Both of the products had similar nutritional and energy values and low ω-3 oil content (1.2-1.6 g ω-3 per kg of food), with an ω-6:ω-3 ratio of 11:1. The rice/chicken dry food contained no wheat, no soy, no beef, no preservatives, no artificial colors and could be used for dogs sensitive to food proteins and/or additives. Neither food contained ingredients have been found to have positive effects on OA.

The supplement used in this study was a pharmaceutically cleaned dense deep sea fish oil (Doils® joints; Nutraceuticoils, Belgium) with low water content and added vitamin E. The fish oil contained 450 mg EPA, 100 mg DHA and 27 mg eicosatetraenoic acids (ETA) per ml, or a total of 63.6% ω-3 s. The fish oil product was supplied by the manufacturer together with a placebo corn oil with fish smell in identical containers. The placebo (corn oil) contained no EPA, DHA or DPA, and only 1% of other ω-3 s (mainly ALA) (Table 4). The products were organized into sequentially numbered treatment units by a research assistant who was not involved in the rest of the study. The dose was the same for all dogs regardless of group; 1 ml of fish or corn oil per 5 kg of dog body weight (BW).

**Table 4:** Fatty acid composition of the supplements and the feed

**Fatty acid**	**Fish oil g/100 g**	**Corn oil g/100 g**	**Food g/100 g**
**Omega-6**	**4.60**	**51.80**	**1.78**
18:2n-6 (LA)	1.30	51.80	1.74
20:4n-6 (AA)	2.10	0.00	0.00
**Omega-3**	**63.60**	**1.00**	**0.16**
18:3n-3 (ALA)	0.60	1.00	0.15
20:5n-3 (EPA)	43.90	0.00	0.00
22:5n-3 (DPA)	2.20	0.00	0.00
22:6n-3 (DHA)	11.30	0.00	0.00
**Other**			
16:0 (sat.)(PA)	0.60	10.23	2.48
16:1n-7(PO)	0.20	0.10	0.33
18:0 (sat.)(ST)	4.60	1.80	1.13
18:1n-9 (OL)	9.70	28.60	4.06
**Total PUFA**	**69.50**	**52.80**	**1.96**
**Total MUF**	**16.40**	**29.50**	**4.50**
**Total SAF**	**6.40**	**12.80**	**3.86**
**Total unknown FA**	**2.50**	**0.00**	**0.00**

*LA* = Linolenic acid, *DPA* = Docosapentaenoic acid, *PA* = Palmitic acid, *PO* = Palmitoleic acid, *ST* = Steric acid, *OL* = Oleic acid, *PUFA* = polyunsaturated fatty acids, *MUF* = Monounsaturated fat, *SAF* = Saturated fat. For rest of the FA nomenclature, see abbreviations.

### Owner assessment

The owners answered multi-dimensional questionnaires about their dogs at seven different times: Six weeks before the first screening visit (W_−8_ = 8 weeks before baseline), during the screening visit (W_−2_ ), at the baseline visit (W_0_), at home once a month between visits (W_4_, W_8_, and W_12_) and at the end of trial-visit (W_16_). The first W_−8_ questionnaire was either downloaded from the Internet or sent to the owners and thereafter returned to us for inclusion consideration. The second and third questionnaires were also pre-treatment baseline questionnaires, completed at the first two visits (pre-baseline screening W_−2_ and baseline W_0_). The fourth to sixth questionnaires were given to the owners at the hospital at W_0_, to be completed at home and sent back in pre-stamped return envelopes. The last questionnaire was completed at the hospital, just before the “end of trial”-visit (W_16_).

The first questionnaire included descriptive questions such as gender, age, diagnose, used treatments etc. and these were only asked once. The following three parts were then included in every round of data collection: (i) Of a total of 19 pain related descriptive scale (0–4) questions on attitude, behaviour, and locomotion, 11 questions formed the owner-assessed HCPI, as described previously [44-47]. (This index now has a new English translation that is closer to the Finnish original; the HCPI-E2, but the validated original remains the same [47,48]). (ii) Two 10-cm VAS: one for lameness and the other for quality of life (QOL). The end of the VAS lines to the left represented no lameness whatsoever and the best possible QOL and to the right, the worse possible lameness or the worse possible QOL. (iii) A question about given NSAIDs or adjuvant analgesics before the trial start or about given rescue medication during the trial used the following scale: “during the last four weeks analgesia was given 1 = not at all, 2 = 1–2 times per 4 weeks, 3 = about once a week, 4 = about 3–5 times a week, 5 = daily/almost daily”.

All questionnaire data collected after the start of trial (W_4_ to W_16_) included additional parts: (iv) Questions about possible adverse reactions to treatment; change in appetite, vomiting, diarrhoea and atopic skin reactions and (v) 18 comparative questions summed together under three categories: “locomotion”, “every-day situations” or “skin & hair coat”. These questions all used a standard 5-point relative response: “Compared to before the beginning of this trial, the dog’s (item) is now. 1 = much better, 2 = a bit better, 3 = the same, 4 = a bit worse, 5 = much worse”. At the end of trial visit the owners also answered questions about (vi) how well the treatment corresponded to their idea of a successful treatment; 1 = corresponded fully, 2 = quite well, 3 = to some parts, 4 = not so well, 5 = not at all, and (vii) if they thought their dog had been in the 1 = treatment or in the 2 = placebo group.

### Veterinary evaluation

At the screening visit (W_−2_) a more thorough clinical, orthopaedic, and neurological examination was performed for inclusion or exclusion by an orthopaedic surgeon specialized in canine pain. The same surgeon made a basic clinical, orthopaedic and neurological evaluation and assessed lameness, jumping, and walking stairs at W_0_ and W_16_, using 0–4 descriptive scales that then were summed to a continuous vet-assessment score with a minimum of 0 (no difficulties whatsoever) and a maximum of 12 (more or less non-ambulatory).

### Gait analysis

Gait was analysed using a force platform at W_0_ and W_16_. The force platform gait analysis (Kistler force platform, Type 9286, Kistler Instrumente AG Winterhur, CH-8408, Switzerland) assesses weight bearing of two limbs at the time. The force platform was submerged into the concrete floor so that the platform and floor surfaces were on the same level. The floor was then covered with a 5-mm-thick rubber mat that extended from 7 m before to 7 m after the platform, forming a 14-m walkway. A hole was cut in the mat over the force platform and a 3-mm gap was left between the force platform mat and the rest of the mat. The signal from the platform was processed and stored using a computer-based software program, and velocities and acceleration were determined by three photoelectric cells placed exactly 1 m apart and a start-interrupt timer system (Aquire 6.0, Sharon Software Inc., DeWitt, USA).

Dogs guided by their owners trotted over the walkway from right to left. The speed was one comfortable for each dog in trot and had to be in the same range (± 0.5 m/s) for the dog each time the test was performed. Acceleration/braking was kept < 0.5 m/s/s and contact had to be made with the platform first by the forelimb and shortly after with the hind limb of the same side for the evaluation to be valid. Three valid measurements for each side and for each visit were then chosen by a blinded assistant according to speed, acceleration, and with no interferences, such as gait abnormalities or extra body movements. These ground reaction forces were normalized for each dog’s body weight and mean peak vertical force (PVF) and mean vertical impulse were used as variables [49].

Only measurements from the most severely affected leg at baseline (W_0_), based on clinical evaluation and confirmed by giving the lowest PVF output, were used in both analyses.

### Blood samples

Blood samples were collected from the dogs at each visit. To test inclusion at the pre-baseline screening visit (W_−2_) creatinine, glucose, serum alanine aminotransferase, alkaline phosphatase, total protein, and cholesterol were analyzed. When cholesterol was high, T4 and TSH were also analyzed. At baseline (W_0_) and at the end of the trial (W_16_) blood urea nitrogen, creatinine, glucose, serum alanine aminotransferase, alkaline phosphatase, total protein, albumin, cholesterol, triglycerides, and fatty acids were analyzed. The total fatty acid analyses, gene-expression analyses and parameters of inflammation and oxidative stress will be reported separately.

### Outcome variables

Our four main outcome variables were force platform variables PVF and impulse, the validated HCPI and the use of rescue NSAIDs. The six secondary outcome variables were owners’ locomotion visual analog scale (VAS), quality of life (QOL-) VAS, comparative questionnaires, the veterinary assessment score, final assessment of outcome and guessing of group.

### Statistical analysis

The number of dogs needed in each group was calculated for a two-tailed test (Fisher) based on a human trial of fish oils for rheumatoid arthritis [10] and a canine OA study [50], giving 32 and 21 dogs per group, respectively, when using statistical power of 0·8 and allowing for a 5% alpha error. As there often is at least 10-20% fallout it was decided to start with 35–40 dogs per group.

Change from baseline measurements to end of trial were used as the response in all of the fitted models, excluding use of rescue NSAIDs. Covariates possibly influencing the outcome were determined beforehand. These 14 variables included age, sex, bodyweight at baseline, used diet, use of pain medication before the study, duration of symptoms, HCPI-score at baseline, body score condition, sterilization/castration and radiology evaluations of hip, knee, back, elbow and lumbo-sacral area.

As the weight of the dog is essential in evaluating change in force platform variables a percentage change from baseline was used as a response in the statistical modeling.

The force platform variables were analyzed using ANOVA and ANCOVA-models. The effects of the covariates were evaluated with ANOVA-models, where only the covariate at issue was used as an explanatory variable. The group effects were evaluated with ANCOVA-models, where the explanatories consisted of treatment group and the significant covariates were modeled ahead.

With HCPI-score direct change from baseline was used as the response and was modeled with a RMANCOVA-model. First the effects of the covariates were modeled with RMANCOVA-models, where the explanatories consisted of the covariate in issue, visit and the baseline-value of the response. Patient was used as a random variable. The same kind of modeling was then constructed for determining the treatment group differences. The model included treatment group, visit, baseline-value of the response, an interaction of group and visit and the possibly significant beforehand evaluated covariates as fixed factors and patient as a random factor. Pair-wise comparisons were calculated from the model for interesting comparisons.

Differences in the use of rescue NSAIDs were evaluated with subject specific cumulative logit-models for repeated measures. The modeling was done for a re-classified variable (3 classes), because the frequency in the higher classes was limited. Again the effects of the covariates were modeled first. The fitted cumulative logit-model had visit, use of NSAIDs 8 weeks before the study and the covariate at issue as fixed factors and patient as a random factor. Because of the same reason of too few answers per values, previous use of NSAIDs was also recoded to only three classes, so that classes “Never” and “1-2 times a month” as well as classes “3-5 times a week” and “Almost daily” were put together. Class “Once a week” remained unchanged. The differences between treatment-groups were evaluated with the same kind of cumulative logit-model, where group, visit, use of NSAIDs eight weeks before the study, interaction of group and visit and possibly significant covariates were used as fixed factors and patient as a random factor. As the owners had been asked to leave out NSAIDs at baseline, the time point W_−8_ was here used as “baseline”, to which the end of trial was compared to.

Five of the secondary outcome variables were modeled with RMANCOVA-models (excluding veterinary assessment score). The same kind of modeling was applied here as with the HCPI-score. Veterinary assessment score was analyzed with an ANCOVA-model, as the assessment had been conducted only at baseline and in the end of study. The same kind of model was applied to the veterinary assessment score as to the force platform variables.

The owners’ final thoughts on the trial as well as the fatty acid concentrations in the serum samples at W_0_ and W_16_ were compared between groups using the independent t-test. Changes in fatty acid concentrations within the groups were calculated using the Wilcoxon rank test.

P-values < 0.05 were considered to be statistically significant and significance levels two-sided. All statistical analyses were done using two different software (SAS® System for Windows, version 9.2, SAS Institute Inc., Cary, NC, USA and SPSS 18.0 for Windows, SPSS Inc. Chicago, IL, USA).

## Abbrevations

AA: Arachidonic acid, an ω-6 FA; ALA: α-linolenic acid, an ω-3 FA; ANCOVA: Analysis of covariance; ANOVA: Analysis of variance; BW: Body weight; CHD: Canine hip dysplasia; CONSORT: Consolidated standards of reporting trials; DHA: Docosahexaenoic acid; DPA: Docosapentaenoic acid, an ω-3 FA; ED: Elbow dysplasia; EFA: Essential fatty acids; EPA: Eicosapentaenoic acid; ETA: Eicosatetraenoic acid, an ω-3 FA; FA: Fatty acid; HCPI: Helsinki Chronic Pain Index; MMP: Matrix metalloproteinase; NSAID: Non-steroidal anti-inflammatory drug; OA: Osteoarthritis; ω: Omega; PVF: Peak vertical force; QOL: Quality of life; RA: Rheumatoid arthritis; RCT: Randomized clinical trial; RMANCOVA: Repeated measures analysis of variance; SD: Standard deviation; TIMP: Tissue inhibitor metalloproteinase; VAS: Visual analog scale; W_-x_: x weeks before baseline; W_0_: Baseline; W_x_: x weeks after baseline.

## Competing interests

The author(s) declare that they have no competing interests. The study protocol was written at and accepted by the department of Equine and Small Animal Clinical medicine, University of Helsinki, Finland. The corresponding author / main investigator (AHB) proposed the study to the company Nutriceuticoils and was not compensated for the work. The fish oil manufacturing company paid the dogs’ radiographs and blood samples for inclusion, the serum fatty acid analysis before and after the trial, the dogs’ foods, the treatment units to be tested and their identical placebo. The doing, ordering, paying and distributing of the whole project were taken care of by the University and the main investigator in Finland, but the company provided the randomized treatment units. Nutriceuticoils had no input on study design, data collection or analysis, on the interpretation of the data, on the decision to write and submit the paper, of the cost of article processing nor on its content. The decision to write and submit the paper for publication was the sole responsibility of the corresponding author. No medical writer was involved in the present paper but the statistical analyzes were bought from a Finnish statistical firm, specialized in clinical trials (4Pharma Ltd.).

## Authors’ contributions

AHB carried out the planning of the study, organized the finances and the research agreement, carried out most of the data collection, evaluated the dogs and wrote the primary draft of the manuscript. JR was present at all of the evaluations, put in all the data into the database, chose the force platform runs and took radiographs and blood samples together with students. AL evaluated all of the dogs’ radiographs. JJ made all of the statistical analyzes through his work at 4Pharma Ltd. OLV approved the study and made major contributions to the manuscript. All authors read and approved the final manuscript.

## Authors’ information

AHB did her PhD on dogs suffering from OA using non-pharmaceutical treatments, often nutraceuticals. She has also validated an owner questionnaire for chronic pain assessment in dogs. She is now an assistant professor in small animal surgery and head of the pain and rehabilitation clinic at the department of Equine and Small Animal Clinical medicine, University of Helsinki, Finland, where she has continued the same line of work. This is the fourth larger RCT where she has been the main investigator and project manager. JR does her PhD on relationships between gene expression and nutrition. AL works at the radiological department at the University of Helsinki. JJ is a medical statistician at a Finnish statistical firm, specialized in clinical trials (4Pharma Ltd.). Outi Laitinen-Vapaavuori is the Professor of Small Animal Surgery at the University of Helsinki.
